# Clinical and histopathological features of chronic hepatitis B virus infected patients with high HBV-DNA viral load and normal alanine aminotransferase level: A multicentre-based study in China

**DOI:** 10.1371/journal.pone.0203220

**Published:** 2018-09-04

**Authors:** Yu-Feng Xing, Da-Qiao Zhou, Jing-Song He, Chun-Shan Wei, Wei-Chao Zhong, Zhi-Yi Han, De-Ti Peng, Mu-Min Shao, Tung-Ting Sham, Daniel Kam-Wah Mok, Chi-On Chan, Guang-Dong Tong

**Affiliations:** 1 Department of Hepatology, Shenzhen Traditional Chinese Medicine Hospital, Shenzhen, China; 2 Department of Pathology, Shenzhen Traditional Chinese Medicine Hospital, Shenzhen, China; 3 Department of Applied Biology and Chemical Technology, The Hong Kong Polytechnic University, Hong Kong, China; 4 State Key Laboratory of Chinese Medicine and Molecular Pharmacology (Incubation), Shenzhen, China; Centre de Recherche en Cancerologie de Lyon, FRANCE

## Abstract

**Background:**

The aim of this study is to reveal the clinical and histopathological features of HBsAg-positive and HBeAg-positive chronic hepatitis B infected patients with high level of HBV DNA, from 17 hospitals and medical centres in China, with alanine aminotransferase levels within the lower region of normal range versus those with levels within the upper region of normal range and to investigate the clinical risk factors for the requirement of treatment through the examination of liver biopsy.

**Methods:**

Liver biopsy was performed on high level of HBV DNA of 455 patients with HBsAg-positive and HBeAg-positive chronic hepatitis B infection and persistently normal alanine aminotransferase level. Liver necroinflammation and fibrosis were graded per the Knodell histological activity index and Ishak’s fibrosis score, respectively. Univariate analysis of the clinical parameters versus necroinflammation and fibrosis was carried out.

**Results:**

Of the subjects in this multicentre-based study, 5.49% and 10.11% had significant necroinflammation with Knodell histological activity index ≥ 9 and hepatic fibrosis stages with Ishak scores ≥ 3, respectively. The subjects were stratified into three age groups (30–39, 40–49 and ≥ 50 years), and our data clearly suggested that age, particularly in the age group over 50, was an independent predictor of liver necroinflammation and fibrosis. Lower HBV-DNA viral levels were found in patients with Knodell histological activity index ≥ 9 or advanced fibrosis (Ishak scores ≥ 3).

**Conclusion:**

Our results showed that histological changes in liver tissues were observed in a significant proportion of patients with persistently normal alanine aminotransferase level. According to the data evaluation results, liver biopsy is advisable for HBeAg-positive chronic hepatitis B infected patients aged older than 40 and high HBV-DNA viral load in China.

## Introduction

Hepatitis B virus (HBV) affects approximately 350 million people worldwide and remains a global public health concern [1, 2]. HBV infection, which prevalence is increasing, is a dynamic process, and the development of HBV is affected by the individual’s immune response and HBV replication. Disease progression is divided into multiple phases, based on the levels of serum hepatitis B envelope antigen (HBeAg), HBV DNA and alanine aminotransferase (ALT) and on the degree of liver inflammation [3]. The impacts of HBV infection can be lifelong as the disease can become chronic and develop further into cirrhosis, liver failure and hepatocellular carcinoma (HCC). The number of HBV-associated deaths due to cirrhosis and/or HCC is more than one million per year [1, 2].

The regions of highest prevalence for HBV infection are Africa, the Pacific region and Asia, where the virus is acquired mainly through perinatal transmission. In China, the prevalence of hepatitis B surface antigen (HBsAg) is high and affects 8.5–10.5% of the adult population [4]. The management of HBV in China is a complex issue, with various intermingled clinical, economic, social and cultural aspects. Since 1992, great efforts have been made by the Chinese government to control HBV infection, including universal HBV vaccination programmes and prescriptions of effective anti-viral drugs [5]. However, adults over 25 years of age are still at high risk as they are not protected by vaccination. It is estimated that the socioeconomic burden will continue to increase over the next two decades, before effective prevention and HBV treatments are applied to most of the population [6].

The primary objective for the management of chronic HBV is to identify patients with an HBV disease that is progressing to more severe liver complications such as cirrhosis and hepatocellular carcinoma and provide these patients with anti-viral treatment. An elevated ALT level is usually an indicator of serious liver damage. However, many studies have suggested that ALT is not an effective marker. One study reported that marked fibrosis was observed in HBV-infected patients (approximately 40%) with the persistently normal (PN) ALT level [7]. Another study conducted in Shanghai suggested that only 38.5% of PNALT chronic HBV infected patients had normal liver histology. The authors noticed that the PNALT HBV patients in the lower half of the normal ALT range had a significantly lower prevalence of histopathology [8]. However, all of these studies were single-centre trials. A multicentre study with more subjects would be valuable to confirm these findings and our current understanding of the development of this important disease.

In the present study, we aimed to examine the histopathological features of high level of HBV DNA HBeAg-positive patients with ALT levels in the lower region of normal (LRN) range and those with ALT levels in the upper region of normal (URN) range by investigating a large cohort of Chinese patients enrolled at 17 hospitals and medical centres. HBV DNA is a quantitative virologic marker reflecting HBV replication level. Evidence supported that elevated serum HBV-DNA level during follow-up is a strong risk predictor of hepatocellular carcinoma independent of HBeAg, serum ALT level, and liver cirrhosis [9]. Univariate and multivariate analyses of the clinical parameters and histological changes in liver tissues were conducted to identify the biochemical parameters that were correlated to the histopathological changes observed in high level of HBV DNA HBeAg-positive patients. This study not only lets us better understand the progression of chronic HBV diseases and to develop prevention and treatment strategies in the future, but it also helped investigate the clinical risk factors for the requirement of treatment through the examination of liver biopsy.

## Material and methods

### Ethics statements

The study was reviewed and approved by the Ethics committees of the Shenzhen Traditional Chinese Medicine Hospital and was registered in the Chinese Clinical Registry (ChiCTR-TRC-09000425). Written informed consent was obtained from the patients included in the study prior to participation.

### Study population

HBeAg-positive patients with chronic HBV infections were recruited from May 2009 to May 2010 at 17 different hospitals and medical centres for this study, and the baseline demographic and laboratory characteristics are listed in Table 1. A total of 455 subjects were recruited, and liver biopsy and routine serum biochemistry tests were conducted for all subjects. The recruitment criteria were as follows: serum HBeAg >1 S/CO, HBsAg > 10IU/mL and < 10^5^ IU/mL, and HBV DNA > 10^5^ IU/mL and < 10^9^ IU/mL with PNALT levels < 40U/L at least 3 times within the year between the ages of 30 and 65. Patients with the following conditions were excluded: 1) co-infection with hepatitis A, C, D, E or G virus; 2) metabolic syndrome or autoimmune hepatitis; 3) alcoholic hepatitis; 4) antiviral drugs taken 6 months before enrolment; 5) abnormal ALT and aspartate aminotransferase (AST) levels within a year before enrolment; 6) cirrhosis; 7) pregnancy or lactation; and 8) serious mental diseases. In some centres, detection of HBV DNA level higher than 10^9^ IU/mL was not accurate, thus, patients with very high viral load were excluded from the study to secure the consistency of the data. All subjects were participated voluntarily and provided informed consent.

**Table 1:** Hospitals or medical centres that participated in this study.

Name	Location (City and Province)
**Shenzhen Traditional Chinese Medicine Hospital**	Shenzhen, Guangdong
**The Third People's Hospital of Shenzhen**	Shenzhen, Guangdong
**The Third Affiliated Hospital, Sun Yat-sen University**	Guangzhou, Guangdong
**The Traditional Chinese Medicine Hospital of Guangdong, Zhuhai Hospital**	Zhuhai, Guangdong
**The Traditional Chinese Medicine Hospital of Guangdong Province**	Guangzhou, Guangdong
**Foshan Traditional Chinese Medicine Hospital**	Foshan, Guangdong
**First Teaching Hospital of Tianjin University of Traditional Chinese Medicine**	Tianjin
**302 Military Hospital of China**	Beijing
**Beijing Ditan Hospital, Capital Medical University**	Beijing
**Xiyuan Hospital CACMS**	Beijing
**Beijing You An Hospital, Capital Medical University**	Beijing
**West China School of Medicine /West China Hospital, Sichuan University**	Nanchong, Sichuan
**Chengdu University of Traditional Chinese Medicine Hospital**	Chengdu, Sichuan
**Shuguang Hospital, Capital Shanghai University of Traditional Chinese Medicine**	Shanghai
**The Second Military Medical University of ChangHai Hospital**	Shanghai
**The Second Affiliated Hospital of Zhejiang Chinese Medical University**	Hangzhou, Zhejiang
**Taian Traditional Chinese Medicine Hospital**	Taian, Shantung
**Wuhan Medical and Treatment Center**	Wuhan, Hubei

### Liver biopsy

All subjects underwent percutaneous liver biopsy guided by ultrasonography. Liver biopsy was performed using 16-G tru-cut biopsy needles (Menghini, Bard Company of America). A minimum of 1.5 cm of liver tissue with at least 4 portal tracts was required for appropriate diagnosis. The specimens were immediately fixed, paraffin-embedded, stained with haematoxylin-eosin and sent to the Department of Pathology at the Shenzhen Traditional Chinese Medicine Hospital. The Knodell histological activity index (HAI) and Ishak’s system were used by two experienced pathologists, who were blinded to the clinical information of the subjects, to grade the collected samples. The Knodell HAI was used to describe the hepatocellular necroinflammation activity with grades of 0–4, while liver fibrosis was semi-quantitatively assessed according to Ishak’s system and was graded from stage 0 to stage 6.

### Serum biochemistry test

All subjects underwent complete blood counts and serum biochemistry detections including ALT, AST, platelet (PLAT), γ-glutamyltransferase (GGT), blood urea nitrogen (BUN) and creatinine (Cr) tests with the Cobas ISE 800 Chemistry Analyzer (Roche Diagnostics, Holliston, MA, USA). HBsAg, hepatitis B surface antibody (HBsAb), HBeAg, hepatitis B envelope antibody (HBeAb) and hepatitis B core antibody (HBcAb) were measured with the Architect i2000 assay (Abbott Laboratories, Philippines). Serum HBV DNA levels were quantified using the COBAS Taqman assay (Roche Diagnostics, Branchburg, NJ, USA), with the lowest detection limit at 12 IU/mL.

### Statistical analysis

All data analyses were performed using SPSS 16.0 software (SPSS Inc., Chicago, IL, USA). Categorical data and gender were expressed as the count (*n*) with percentages. Continuous data were presented as the mean and standard deviation (mean ± SD). HBV DNA level was log-transformed due to their skewed distributions. Differences in continuous variables between groups were tested with the independent samples *t*-test with Levene's test for equality of variances or one-way analysis of variance (ANOVA). For one-way ANOVA, Tukey’s *post hoc* test was used after test on the homogeneity of the variances. A *p* value (2-tailed) < 0.05 was regarded as statistically significant. Multivariate (binary) logistic regression analysis was used to find the determinants for prediction of significant fibrosis (Stage ≥ 3) and necroinflammation (HAI score ≥ 9).

## Results

### Patient characteristics

A total of 455 patients were enrolled in this study; 287 (62.53%) were male. The baseline characteristics of all patients are summarized in Table 2. The mean age was 34.83 years (range, 30–63 years), and the average Body Mass Index (BMI) was 22.24 ± 5.96 kg/m^2^. The HBV DNA levels ranged from 4.14 to 10.94 (in log_10_ IU/mL), while the HBsAg level ranged from 2.08 to 5.38 (in log_10_ IU/mL). The patients were subcategorized into two subgroups: URN ALT (ALT ≥ 30U/L for males or ≥ 19U/L for females) and LRN ALT (ALT < 30U/L for males or <19U/L for females), according to the guidelines of the American Association for the Study of Liver Diseases (AASLD) [10].

**Table 2:** Demographic and laboratory characteristics of the present cohort study.

**Parameters**	**Mean ± SD, or *n* (percentage)**
**Age in years**	34.86 ± 6.38
**30–39**	372 (81.76%)
**40–49**	64 (14.07%)
**≥50**	19 (4.18%)
**Gender, *n* (%)**	
**Male**	287 (63.08%)
**Female**	168 (36.92%)
**BMI (kg/m^2^)**	22.23 ± 5.97
**Geographical distribution**	
**Eastern, China**	92 (20.48%)
**Western, China**	41 (8.93%)
**Southern, China**	229 (50.33%)
**Northern, China**	46 (10.02%)
**Central, China**	47 (10.24%)
**ALT (IU/L)**	27.29 ± 8.19
**LRN ALT^a^**	189 (41.54%)
**URN ALT^b^**	266 (58.46%)
**AST(IU/L)**	25.01 ± 6.59
**PLAT count (10^9^/L)**	192 ± 52
**GGT (IU/L)**	22.14 ± 10.92
**BUN (mmol/L)**	5.12 ± 2.16
**Cr (μmol/L)**	74.81 ± 15.75
**HBV DNA (log_10_ IU/mL)**	8.14 ± 0.99
**Knodell HAI, score**	
**≥ 9**	25 (5.49)
**< 9**	430 (94.51)
**Ishak’s system, score**	
**≥ 3**	46 (10.11)
**< 3**	409 (89.89)
**Fib-4**	0.19 ± 0.11
**< 1.45**	455 (100)
**APRI**	0.41 ± 0.23
**< 0.5**	338 (74.29)
**> 0.5**	117 (25.71)
**GPR**	0.13 ± 0.08
**< 0.34**	443 (97.36)
**> 0.34**	12 (2.64)

^a^ LRN ALT: ALT < 30U/L (male), ALT < 19U/L (female).

^b^ URN ALT: ALT ≥ 30U/L (male), ALT ≥ 19U/L (female).

### Prevalence of significant liver histopathology

**Fig 1:**
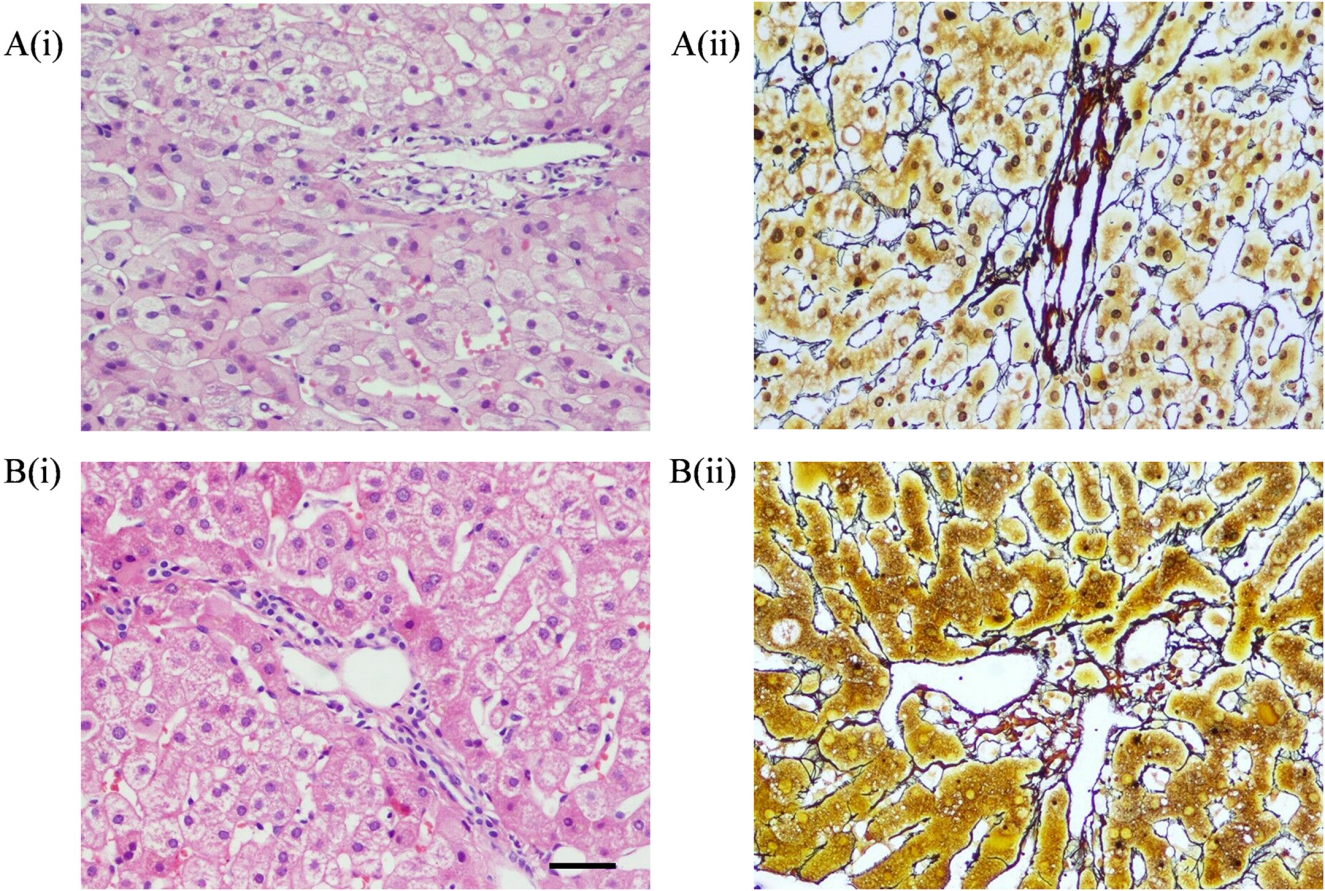
Representative liver biopsy photomicrographs with haematoxylin and eosin staining and view at a magnification of 100×. Bar = 100 μm. A: LRN ALT group, i) Knodell HAI score 1, ii) Ishak score 0; B: URN ALT group, i) Knodell HAI score 2, ii) Ishak score 1.

**Fig 2:**
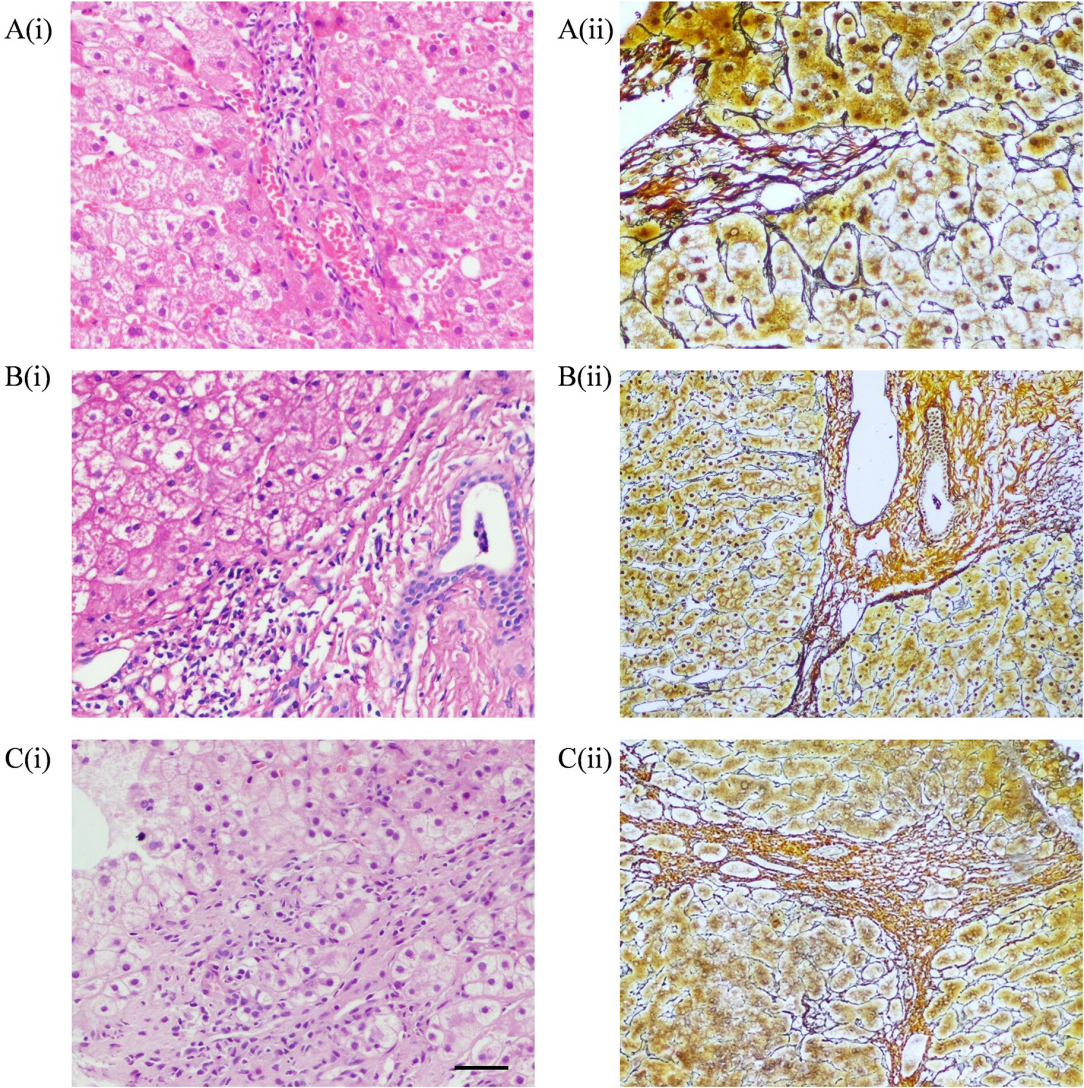
Representative liver biopsy photomicrographs with haematoxylin and eosin staining and view at a magnification of 100×. Bar = 100 μm. A: Age at 30–39, i) Knodell HAI score 3, ii) Ishak score 2; B: Age at 40–49, i) Knodell HAI score 5, ii) Ishak score 4; C: Age ≥ 50, i) Knodell HAI score 10, ii) Ishak score 5.

The histopathological data of the liver biopsy samples are shown in Table 3. The mean Knodell HAI score was 3.56 ± 2.98 (Median 3, range 0–17), while the mean stage was 1.15 ± 1.21 (Median 1, range 0–6). Regarding liver fibrosis, 189 patients were S0 (41.5%), 84 were S1 (18.5%), 136 were S2 (29.9%), 24 were S3 (5.3%), 16 were S4 (3.5%), 5 were S5 (1.1%), and 1 was S6 (0.2%). The majority of patients had minimal histological disease: 69.9% (Knodell HAI grade 0–1) of the patients had no or mild inflammation, while 60% of the patients had stage 0–1 fibrosis. In addition, only 8 patients had significant necroinflammation (HAI score ≥ 9) or fibrosis (stage 4 or above) in the LRN ALT group, while 21 patients had significant necroinflammation (HAI score ≥ 9) or fibrosis (stage 4 or above) in the URN ALT group. Representative liver biopsy photomicrographs with haematoxylin and eosin staining are shown in Figs 1 and 2.

**Table 3:** Histopathological findings in the liver biopsy specimens of chronic hepatitis B patients according to the Knodell HAI and Ishak’s scoring system.

	*n* (percentage)	
	Male (*n* = 287)	Female (*n* = 168)
**Knodell HAI**		
**Grading 0 (0 scores)**	34 (11.8%)	18 (10.7%)
**Grading 1 (1–4 scores)**	178 (62.0%)	88 (52.4%)
**Grading 2 (5–8 scores)**	61 (21.3%)	51 (30.3%)
**Grading 3 (9–12 scores)**	10 (3.5%)	9 (5.4%)
**Grading 4 (13–18 scores)**	4 (1.4%)	2 (1.2%)
**Ishak’s system**		
**Stage 1 (0–1 score)**	177 (61.7%)	96 (57.2%)
**Stage 2 (2 score)**	82 (28.6%)	54 (32.1%)
**Stage 3 (3 score)**	11 (3.8%)	13 (7.7%)
**Stage 4 (4 score)**	12 (4.2%)	4 (2.4%)
**Stage 5 (5 score)**	4 (1.4%)	1 (0.6%)
**Stage 6 (6 score)**	1 (0.3%)	0 (0.0%)

**Fig 3:**
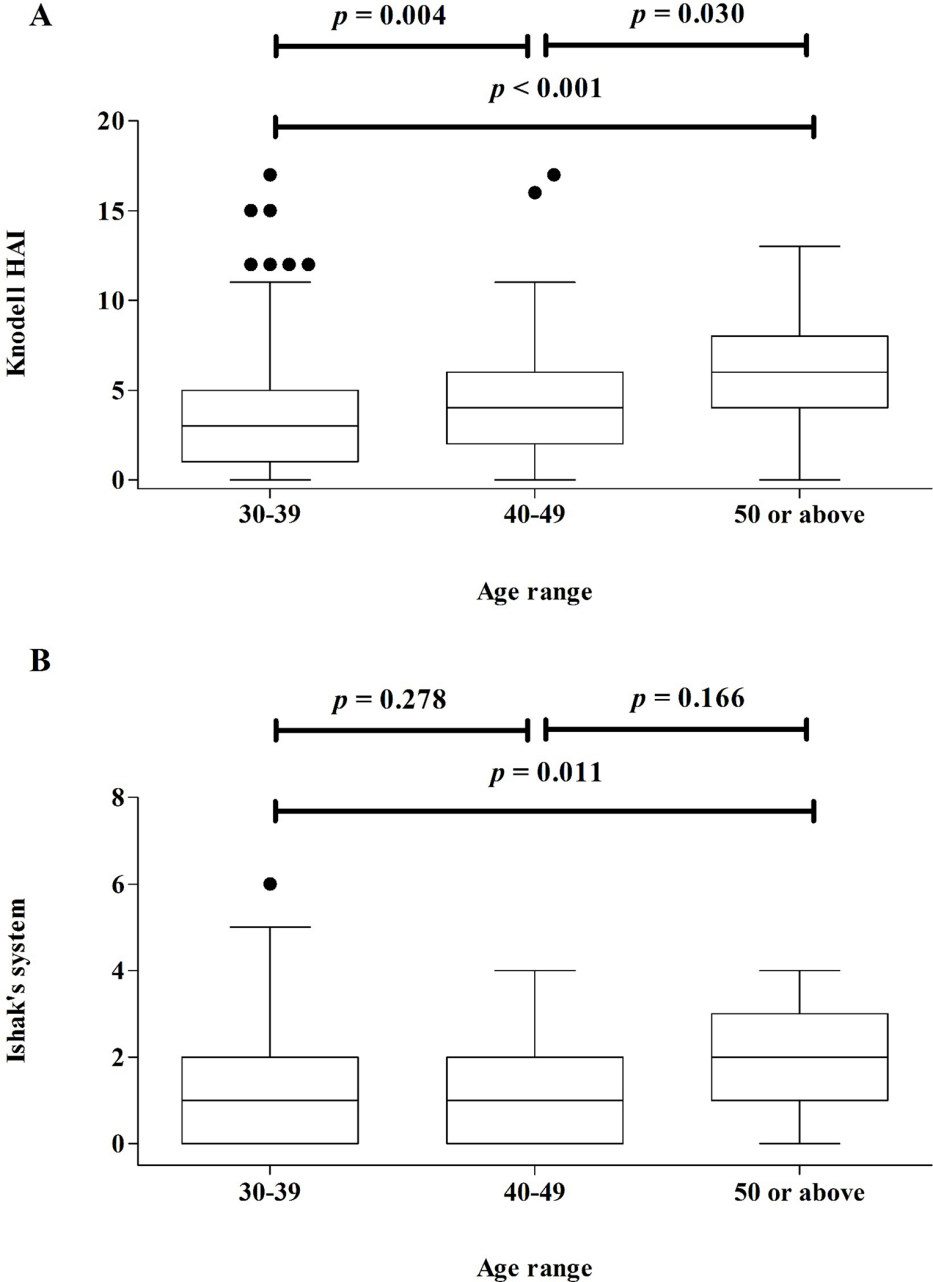
**Prevalence of liver necroinflammation (A) and liver fibrosis (B) among patients classified by age range**.

The distributions of the Knodell HAI score and Ishak’s fibrosis stage between the URN ALT and LRN ALT groups are shown in Table 4. Significant differences in liver necroinflammation and fibrosis (*p* < 0.01) between the groups were observed. The subjects were stratified into three age groups, namely 30–39, 40–49 and ≥ 50, and liver biopsies with marked liver necroinflammation and liver fibrosis in the patients in the different age groups are depicted in Fig 3. Significant differences in liver necroinflammation between the age ranges 30–39 and 40–49 (*p* = 0.004) and the age ranges 40–49 and ≥ 50 (*p* = 0.030) were observed. However, no significant differences in liver fibrosis were evident between the age groups 30–39 and 40–49 (*p* = 0.278) or the age groups 40–49 and ≥ 50 (*p* = 0.166). A significant difference was observed only for liver fibrosis between the age ranges 30–39 and ≥ 50 (*p* = 0.011).

**Table 4:** Univariate analysis of factors between the LRN ALT and URN ALT groups.

Factors	LRN ALT	URN ALT	*p* value of *t* test
	mean ± SD	mean ± SD	
**Age (years)**	34.69 ± 6.52	34.97 ± 6.28	0.638
**Gender**			
**Male (*n*)**	147	140	
**Female (*n*)**	42	126	
**ALT**	20.34 ± 5.43	32.14 ± 6.00	< 0.001
**Necroinflammatory activity**	2.79 ± 2.65	4.11 ± 3.08	< 0.001
**Fibrosis stage**	0.94 ± 1.11	1.30 ± 1.25	0.002
**AST**	20.68 ± 3.95	28.09 ± 6.36	< 0.001
**Fib-4**	0.21 ± 0.09	0.18 ± 0.11	0.010
**APRI**	0.34 ± 0.13	0.47 ± 0.26	< 0.001
**GPR**	0.12 ± 0.07	0.13 ± 0.10	0.086
**HBV-DNA level (Log_10_)**	8.23 ± 0.99	8.08 ± 0.99	0.107

### Comparison of the performances of AST, FIB-4, APRI, and GPR in predicting significant histology

Univariate analysis of the demographic and clinical characteristics of the patients with and without marked hepatic histological changes are summarized in Tables 5 and 6. Patients with significant liver necroinflammation (HAI score ≥ 9) had a significantly higher AST and APRI (AST to PLAT Ratio) value (*p* < 0.001) than the patients without liver necroinflammation (HAI score < 9). Additionally, the values of AST and GPR (GGT to PLAT ratio) in the patients with marked hepatic fibrosis (stage ≥ 3) were increased significantly (*p* < 0.001) compared to the patients without liver fibrosis (stage < 3).

**Table 5:** Univariate analysis of the factors and liver necroinflammatory activity in the PNALT patients.

Factors	Necroinflammatory activity		*p* value of *t* test
	HAI < 9	HAI ≥ 9	
	mean ± SD	mean ± SD	
**Age (years)**	34.70 ± 6.25	37.60 ± 7.92	0.083
**Gender**			
**Male (*n*)**	269	18	
**Female (*n*)**	161	7	
**AST**	24.75 ± 6.55	29.51 ± 5.83	< 0.001
**ALT**	26.96 ± 8.16	31.99 ± 7.35	0.003
**Fib-4**	0.19 ± 0.11	0.24 ± 0.11	0.017
**APRI**	0.40 ± 0.23	0.57 ± 0.22	< 0.001
**GPR**	0.12 ± 0.08	0.17 ± 0.10	0.005
**HBV-DNA level (Log_10_)**	8.187 ± 0.94	7.44 ± 1.47	0.00720

**Table 6:** Univariate analysis of the factors and liver fibrosis stages in the PNALT patients.

Factors	Fibrosis stage		*p* value of *t* test
	Stage < 3	Stage ≥ 3	
	mean ± SD	mean ± SD	
**Age (years)**	34.59 ± 6.15	37.22 ± 7.80	0.032
**Gender**			
**Male (*n*)**	259	28	
**Female (*n*)**	150	18	
**AST**	24.56 ± 6.38	28.74 ± 7.26	< 0.001
**ALT**	26.86 ± 8.23	30.63 ± 7.08	0.003
**Fib-4**	0.19 ± 0.11	0.21 ± 0.10	0.143
**APRI**	0.41 ± 0.23	0.49 ± 0.19	0.011
**GPR**	0.12 ± 0.08	0.17 ± 0.08	< 0.001
**HBV-DNA level (Log_10_)**	8.22 ± 0.96	7.49 ± 1.37	0.001

Multivariate analysis with different clinical factors with and without marked hepatic histological changes are summarized in Table 7. AST, GPR and HBV DNA level were associated with significant liver necroinflammation and fibrosis.

**Table 7:** Multivariate analysis of risk factors for both liver necroinflammatory activity and fibrosis stages in the PNALT patients.

Factors	HAI< 9 and/or Stage < 3	HAI ≥ 9 and/or Stage ≥ 3	Multivariate analysis			
			*p* value	Odds ratio	95% Confidence interval	
**Age**	34.48 ± 6.05	37.79 ± 8.00	0.068	1.08	0.99–1.18	
**Male (*n*)**	256	31				
**Female (*n*)**	147	21				
**AST**	24.52 ± 6.37	28.86 ± 7.08	0.010	1.10	1.02–1.18	
**ALT**	26.75 ± 8.22	30.99 ± 7.01	0.888	0.99	0.88–1.12	
**Fib-4**	0.19 ± 0.11	0.22 ± 0.09	0.527	0.01	< 0.01–9.46 x 10^3^	
**APRI**	0.40 ± 0.23	0.50 ± 0.19	0.971	1.12	< 0.01–427.55	
**GPR**	0.12 ± 0.08	0.16 ± 0.08	0.007	450.23	5.44–3.73 x 10^4^	
**HBV-DNA level (Log_10_)**	8.21 ± 0.91	7.61 ± 1.39	0.002	0.64	0.49–0.86	

### Characteristics of PNALT patients’ liver histopathology in relation to their HBV DNA levels

**Fig 4:**
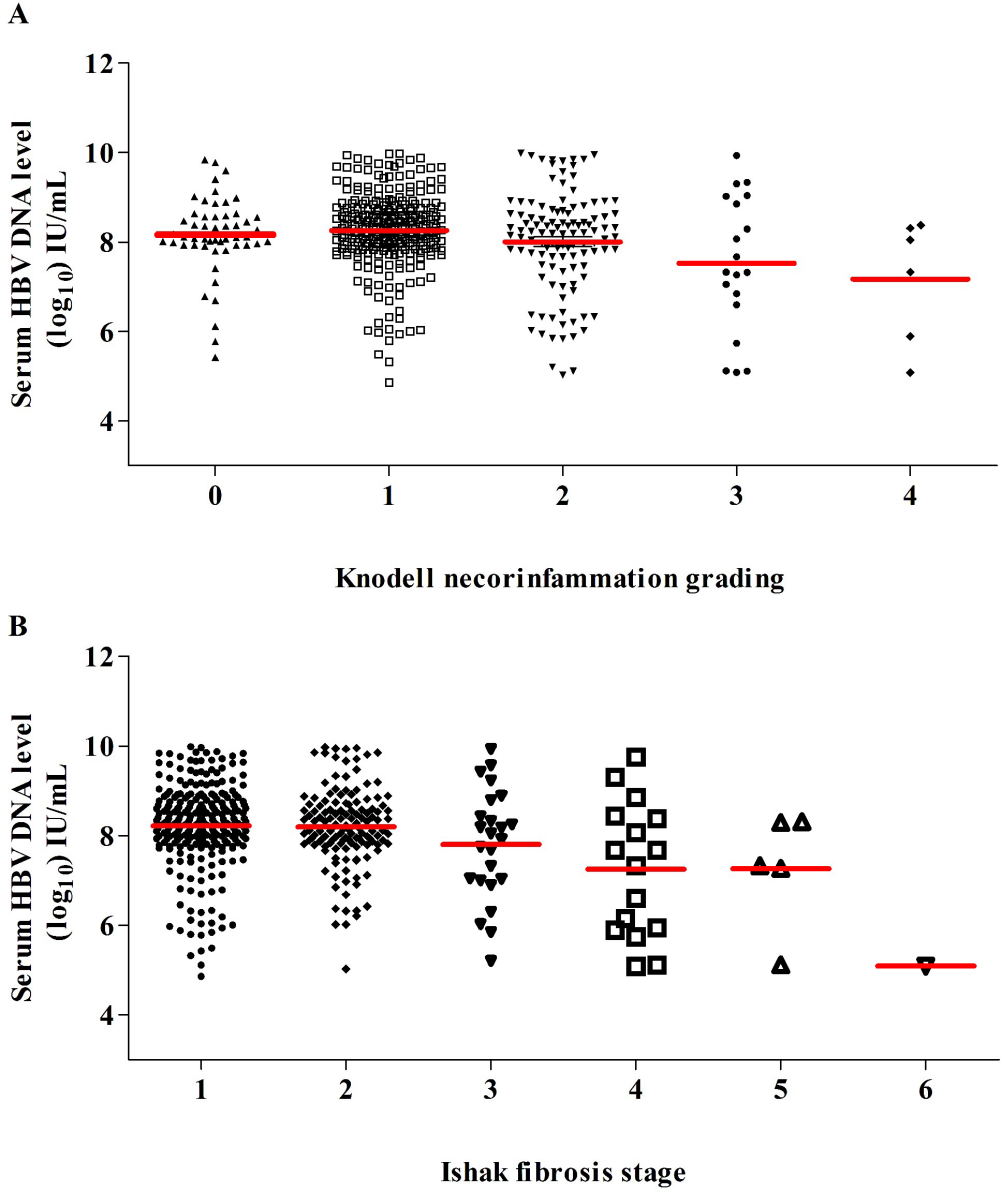
**Serum HBV-DNA levels in different grading of liver necroinflammation (A) and stages of liver fibrosis (B)**.

The distribution of HBV-DNA levels of all patients was evaluated across different grading of liver necroinflammation (Fig 4A) and stages of liver fibrosis (Fig 4B). The HBV-DNA levels displayed a decreasing trend as necroinflammation became serious while similar trends of declined HBV-DNA levels were observed as fibrosis progressed.

## Discussion

Liver biopsy remains the gold standard for assessing the degree of liver injury, including necroinflammatory activity, fibrosis stage, and the presence of other concomitant factors. However, liver biopsy has several drawbacks, such as the typical risks of an invasive method, heterogeneous variability in the sample and a lack of patient acceptance [11], which restricts the frequency and coverage of liver biopsy as a clinical application. The serum level of ALT, a cytoplasmic enzyme in hepatocytes, is usually utilized to reflect hepatocellular damage [12], and nearly all guidelines for treatment of chronic HBV infection use the serum ALT level as a criterion for antiviral treatment [13].

Interestingly, a number of controversial findings about the correlation between the ALT level and liver histology have been reported. Low scores reflecting liver change within the normal ALT range have previously been reported in chronic HBV patients, while significant liver fibrosis and inflammation have been reported in 37% of 192 HBeAg-positive chronic HBV-infected patients with PNALT [14, 15]. Conceivably, the serum ALT level is affected by many factors, including BMI, gender, metabolism abnormalities and geographic origin [16, 17]. Because of their normal ALT levels, these patients will not receive any treatment under the current national guidelines for HBV treatment. Hence, these HBV-infected patients lose the best time window to control of the progress of the disease. Therefore, the identification of patients with PNALT who still suffer from severe liver injury is challenging. Recently, indirect serum markers such as the FIB-4 index, APRI and GPR, which affect the ALT level and other serum parameters, have been proposed as clinical markers in routine laboratory measurements to predict the severity of liver damage [11, 18].

According to the latest version of American Association for the Study of Liver Diseases (AASLD) guidelines, patients with high HBV-DNA level should be monitored at 3—to 6—month intervals and more frequent monitoring should be performed when ALT levels become elevated [10]. Patients with compensated liver disease who remain high HBV-DNA levels after a 3—to 6 -month period of elevated ALT levels greater than 2 times of the upper limit of normal level (>50 U/L for women and >70 U/L for men) should be considered for antiviral treatment. Also, liver biopsy should be considered in patients with persistent borderline normal or slightly elevated ALT levels, particularly in those patients over age 40 who have been infected with HBV from a young age.

In the present study, all patients, who had not join any vaccination programmes before, had persistently normal ALT level despite high HBV-DNA viral load in serum. It is believed that they were one of susceptible high-risk group in China’s population.

All PNALT patients in our study were divided into two subgroups, namely LRN ALT and URN ALT group. Liver biopsies on 455 HBeAg-positive chronic HBV-infected patients with PNALT were carried out, and our data in this multicentre-based study showed that 5.49% and 10.11% of these patients had significant liver necroinflammation with Knodell HAIs ≥ 9 and hepatic fibrosis stages with Ishak scores ≥ 3, respectively. Therefore, a remarkable proportion of Chinese HBV patients with PNALT actually had marked liver damage. Besides, the treatment of chronic hepatitis B adult patients in the immune tolerant phase are controversial and most of current clinical guidelines still recommend that those patients with high HBV DNA levels (typically > 10^6^ IU/mL), positive HBeAg, and ALT (<30 U/L) need to receive antiviral therapy [10]. Our data showed that 3.7% and 7.3% of such patients had significant liver necroinflammation with Knodell HAIs ≥ 9 and hepatic fibrosis stages with Ishak scores ≥ 3, respectively.

These findings, together with previous studies on the liver injuries of PNALT HBV patients, are summarized in Table 8. The Knodell and Metavir scoring systems are commonly used to assess liver necroinflammation, and direct comparisons of the two systems are difficult. Among seven studies carried out in Chinese communities [7, 8, 19–23], except a study in Guangzhou, 13% to 36.5% of PNALT patients had moderate liver necroinflammation. When the studies carried out in Shanghai and Zhejiang are directly compared, the population of PNALT patients with an HAI score ranging from 4 to 6 is increasing. The situation in China aligns with observations in other Asian countries [24–26], in which 35% of PNALT patients with mild and moderate necroinflammation were observed.

**Table 8:** A summary of PNALT in other previous studies [7, 8, 19–26] and this study.

Study location	*n*	Criteria	Dataset in the previous studies		Dataset in this study	
			Proportion		Proportion	
			Necro-inflammation	Fibrosis	Necro-inflammation	Fibrosis
**Shanghai, China [8]**	252	HAI ≥ 4 (0–18)	21.80%	15.90%	36%	10.10%
		Ishak stage ≥ 3 (0–6)				
**Zhejiang, China [19]**	115	HAI ≥ 7 (0–18)	36.50%	15.50%	16.70%	10.10%
		Ishak stage ≥ 3 (0–6)				
**Jiangsu, China [20]**	273	Knodell score ≥ 7 (0–14)	24.50%	4.90%	N/A	N/A
		Knodell score≥ 2 (0–4)				
**Guangzhou, China [7]**	140	Metavir score ≥ A2 (A0-A3)	1.20%	49.40%	N/A	N/A
		Metavir score ≥ F2 (F0-F4)				
**Zhejiang, China[21]**	102	Metavir score ≥ G2 (G0-G4)	24.50%	22.50%	N/A	N/A
		Metavir score ≥ F2 (F0-F4)				
**Xiamen, China [22]**	101	Scheuer’s score ≥ G2 (G0-G4)	12.90%	35.90%	N/A	N/A
		Scheuer’s score ≥ S2 (S0-S4)				
**Jiangsu, China [23]**	288	Metavir score ≥ A2 (A0-A3)	29.20%	25.30%	N/A	N/A
		Metavir score ≥ F2 (F0-F4)				
**India [24]**	189	HAI ≥ 4 (0–18)	36.50%	23.80%	36%	N/A
		Fibrosis stage ≥ 2 (0–4)				
**Thailand [25]**	142	Metavir score ≥ A2 (A0-A3)	13.40%	12.70%	N/A	N/A
		Metavir score ≥ F2 (F0-F4)				
**Turkey [26]**	120	HAI ≥ 6 (0–18)	15%	35.90%	22.60%	40%
		Ishak stage ≥ 2 (0–6)				

N/A, not applicable.

Regarding liver fibrosis, a large variation from 4.9% to 49.4% was reported for PNALT patients with moderate liver fibrosis among seven studies in a Chinese community. A direct comparison of the studies carried out in Shanghai and Zhejiang shows that our dataset is consistent with the previous results and the finding that less than 20% of PNALT patients have moderate liver fibrosis. However, the percentage of PNALT patients with liver fibrosis increases to 40% when the Ishak score is set to stage 2. Thus, a significant population of PNALT patients with moderate liver fibrosis is clearly evident.

The FIB-4 index and APRI, two well-validated parameters for liver fibrosis based on a simple determination of serum biomarkers, were determined and correlated with liver biopsy data. A significant difference (*p* = 0.011) between the patient groups with Ishak ≥ 3 and Ishak < 3 was observed by using APRI for the liver fibrosis stage. At the same time, a significant difference (*p* < 0.001) between the patient groups with Knodell HAI ≥ 9 and HAI < 9 was observed by using APRI for liver necroinflammatory activity. However, the performance of the FIB-4 index as a predictor was not satisfactory. The range of the FIB-4 index among 455 patients was from 0.06 to 1.41, which was under the limit of 1.45. Additionally, there was no significant difference between the patient groups with Ishak ≥ 3 and Ishak < 3. Recently, the GPR index was developed to predict liver fibrosis and cirrhosis in patients with chronic HBV infection in West Africa [27]. Both univariate and multivariate analysis showed that there was a significant difference between the HBeAg-positive chronic hepatitis B infected patients groups with Knodell HAI ≥ 9 & HAI < 9 and Ishak ≥ 3 & Ishak < 3 by using the GPR index for liver necroinflammatory activity and fibrosis. Our results was also in agreement with a previous report describing the diagnosis of fibrosis and cirrhosis in HBeAg-positive HBV-infected Chinese patients [4].

Age is an important factor that affects the histological activity and stage of liver disease. On the basis of the AASLD [10] and Asian Pacific Association for the Study of the Liver (APASL) guidelines [28], HBeAg-positive patients with chronic HBV infections who are over 40 years old are currently considered at risk. In our present study, univariate analysis of liver necroinflammation and fibrosis in the three age subgroups (30–39, 40–49 and ≥ 50 years) was performed. The results clearly demonstrated that age, particularly in the older subgroup, was an independent predictor of significant liver necroinflammation and fibrosis, in those PNALT patients with high HBV-DNA level. In addition, our results also revealed that lower HBV-DNA levels were found in patients with active liver necroinflammation and advanced fibrosis. Serum level of HBV DNA is a dynamic parameter in patients with chronic hepatitis B and is commonly to be used as criterion for antiviral treatment in patients with chronic hepatitis B. Similar study carried out in Taiwan cohort found that participants with persistent elevation of serum HBV-DNA level during follow-up had the highest hepatocellular carcinoma risk [9]. It may imply that active host immunity would be triggered against HBV replication and possibly results in chronic liver injury. Thus, early assessment via liver biopsy is advisable for PNALT patients with persistently high HBV-DNA viral load over 40 years old in China, in addition to regular follow-up blood tests.

There were several limitations that affected the interpretation of our findings. This study was a retrospective cross-sectional study that could not exclude selection bias of patients. Moreover, the number of patients aged ≥ 50 years was relatively small, and follow-up of patients after liver biopsy was insufficient. Findings of this study could not be generally applied to the genotypes other than genotype B.
